# Commercial enzyme-linked immunosorbent assay *versus* polymerase chain reaction for the diagnosis of chronic Chagas disease: a systematic review and meta-analysis

**DOI:** 10.1590/0074-02760150296

**Published:** 2016-01

**Authors:** Pedro Emmanuel Alvarenga Americano do Brasil, Rodolfo Castro, Liane de Castro

**Affiliations:** 1 Fundação Oswaldo Cruz, Instituto Nacional de Infectologia Evandro Chagas, Laboratório de Pesquisa Clínica em Doença de Chagas, Rio de Janeiro, RJ, Brasil; 2 Fundação Oswaldo Cruz, Instituto Nacional de Infectologia Evandro Chagas, Laboratório de Pesquisa Clínica em DST e AIDS; 3 Universidade Federal do Estado do Rio de Janeiro, Instituto de Saúde Coletiva, Rio de Janeiro, RJ, Brasil; 4 Fundação Oswaldo Cruz, Instituto Nacional de Infectologia Evandro Chagas, Laboratório de Farmacogenética, Rio de Janeiro, RJ, Brasil

**Keywords:** Chagas disease, Trypanosoma cruzi, diagnosis, polymerase chain reaction, enzyme-linked immunosorbent assay

## Abstract

Chronic Chagas disease diagnosis relies on laboratory tests due to its clinical characteristics. The aim of this research was to review commercial enzyme-linked immunosorbent assay (ELISA) and polymerase chain reaction (PCR) diagnostic test performance. Performance of commercial ELISA or PCR for the diagnosis of chronic Chagas disease were systematically searched in PubMed, Scopus, Embase, ISI Web, and LILACS through the bibliography from 1980-2014 and by contact with the manufacturers. The risk of bias was assessed with QUADAS-2. Heterogeneity was estimated with the I^2^ statistic. Accuracies provided by the manufacturers usually overestimate the accuracy provided by academia. The risk of bias is high in most tests and in most QUADAS dimensions. Heterogeneity is high in either sensitivity, specificity, or both. The evidence regarding commercial ELISA and ELISA-rec sensitivity and specificity indicates that there is overestimation. The current recommendation to use two simultaneous serological tests can be supported by the risk of bias analysis and the amount of heterogeneity but not by the observed accuracies. The usefulness of PCR tests are debatable and health care providers should not order them on a routine basis. PCR may be used in selected cases due to its potential to detect seronegative subjects.

Chagas disease is a condition in which the infectious agent is a parasite called*Trypanosoma cruzi*. It is considered a neglected disease and typically occurs in poor rural areas of Latin America. Since 2000, it has progressively become of great interest worldwide due to its increasing presence in non-Latin American countries (Savioli & Daumerie 2008, de Andrade et al. 2011, Tanowitz et al. 2011). Infected people who travel from Latin America have been identified as a source of disease transmission through blood transfusion or organ donation in non-Latin American countries (Savioli & Daumerie 2008).

Chronic Chagas disease diagnosis is rather difficult to determine due to two basic clinical issues: (i) a high prevalence of a clinical form at the chronic phase in which there is no target organ findings, the indeterminate form (Ribeiro & Rocha 1998, Prata 2001), and (ii) it is a lifelong condition where the clinical suspicion may come many decades later in infected subjects (Lapa et al. 2012) after exposure when physicians often do not identify a history of exposure or a history of acute illness signs.

Currently, diagnostic investigations of the chronic phase rely on serological tests. There are many recommendations of the number and combinations/algorithms of serological tests to conduct diagnostic investigation in the chronic phase (Albajar et al. 2005, MS/SVS 2005, OPAS 2005, Bern et al. 2007, MINSAL 2007, 2008, de Andrade et al. 2011, MPPS 2014). However, enzyme-linked immunosorbent assays (ELISA), either conventional or with recombinant antigens (ELISA-rec), are often mentioned as a preferred test (MINSAL 2007, 2008). Additionally, some guidelines mentioned that polymerase chain reaction (PCR), combined either in series or in parallel with serological test(s), can be used for chronic Chagas disease diagnostic investigation (Albajar et al. 2005, MS/SVS 2005). Previous systematic reviews of diagnostic tests for Chagas disease concluded that these tests were not well studied, there was a high heterogeneity in their accuracies, and the serological tests’ accuracies are likely overestimated (Brasil et al. 2010, Afonso et al. 2012). The lack of homogeneity of in-house PCR tests’ protocols have also been discussed as a source of heterogeneity of their accuracy (Brasil et al. 2010, Schijman et al. 2011).

Therefore, we updated a previous systematic review on diagnostic tests for chronic Chagas disease (Brasil et al. 2010) and this time focused on commercially available tests. The aim of this research was to systematically review, explore heterogeneity, and summarise the diagnostic test accuracy (sensitivity and specificity) for commercial ELISA tests, commercial ELISA tests with recombinant antigens, and PCR for the diagnosis of chronic Chagas disease when compared to two combined serological tests.

## Eligibility criteria

The abstracts were eligible for full text evaluation if their aims were at least one of the following: (i) to estimate sensitivity or specificity of one or more ELISA or PCR tests for chronic Chagas disease, (ii) to estimate the accuracy of an ELISA or PCR test for chronic Chagas disease, (iii) to test a new ELISA or PCR test for chronic Chagas disease, or (iv) to estimate any validity measure for ELISA or PCR for chronic Chagas disease such as the area under the receiver operating characteristics (ROC) curve or predictive values. If abstracts had unclear objectives but partially met any of the inclusion criteria, or had unclear objectives and had any of the validity measures (as described above) as a result, they were also included for full text retrieval. Abstracts with the following characteristics were not included: (i) not conducted with human volunteers or with samples from human beings, (ii) an indication that the tests were studied in a verification of cure scenario, (iii) the investigations were concerning exclusively acute infection or newborns, or with mixed data from acute and chronically infected patients without the possibility of disaggregation. After full text retrieval, the following inclusion criteria were applied for quality evaluation and data extraction: (i) the investigations should be original (narrative reviews, editorials, or letters without primary data were excluded), (ii) they should be quantitative investigations, (iii) every investigation must have two samples (1 representing those with chronic Chagas disease and another representing those without chronic Chagas disease), (iv) they must have results with enough data to allow extraction (or calculation) of true positives, false negatives, false positives, and true negatives of each test, and (v) they must not involve strictly laboratory validation research.

Only texts published after 1980 were included. Although only abstracts in English, Spanish, or Portuguese were obtained, no language restriction was applied to the full text evaluation.

Diagnostic studies from Phase 1 to Phase 3 were included. Phase 1 studies are case-controls studies where the definitions of the cases and controls are not necessarily defined by the same reference. Phase 2 studies are case-control studies where the same reference for cases and controls are strictly the same and indeterminate subjects are usually discarded. Phase 3 studies are cross-sectional or diagnostic cohort studies with the consecutive inclusion of subjects in which the suspicion of the condition of interest is the main inclusion criteria.

## Information sources

Data from a previous systematic review was used (Brasil et al. 2010) and updated. The previous systematic review included data from 1980-2009 and the update period included data from 2009-May 2014. Nevertheless, reviewers filled the new forms with data extracted again from all of the full texts from the previous systematic review. The main difference in the version of the forms was the risk of bias section.

The Brazilian Health Surveillance Agency (ANVISA) (the regulatory agency for health products) website was visited up to 10 October 2014 to find possible tests of interest for this research. Moreover, authors visited the manufacturer’s website up to 10 October 2014 to find technical reports, test brochures, or set of studies’ results from tests accuracy data. In addition, the authors tried electronic mail contact with manufacturers of the tests registered at ANVISA’s website to request this same information.

The authors’ search continued up to 31 March 2014 and included the following databases for abstracts of interest: PubMed/MEDLINE, Scopus, Embase, LILACS and ISI Web. A search of the bibliography of each full text retrieved was conducted while the full texts were being evaluated.

## Search strategy

The following search terms were used at Medline/PubMed: “Chagas Disease” (MeSH) OR “Trypanosoma cruzi” (MeSH) AND ELISA OR enzyme AND linked AND assay, OR PCR OR polymerase AND chain AND reaction, AND sensitive* (Title/Abstract) OR sensitivity and specificity (MeSH Terms) OR diagnos* (Title/Abstract) OR diagnosis (MeSH:noexp) OR diagnostic* (MeSH:noexp) OR diagnosis, differential (MeSH:noexp) OR diagnosis (Subheading:noexp) OR “Reproducibility of Results” (MeSH) OR reliability OR reproducibility.

The search strategies used in the remaining databases were adapted from the one above and can be accessed at protocol registration at: crd.york.ac.uk/PROSPERO/display_record.asp?ID=CRD42014005733.

## Study records

The retrieved abstracts were stored in a reference manager library. The library was split in two sets. Both sets were classified by one of the reviewers/authors and the remaining authors classified one set each. The reviewers independently tagged each abstract as eligible or not eligible, and later the reviewers met and compared each of their classifications and solved their discrepancies. For each eligible abstract, the full text was retrieved and the same independent classification was conducted. For each elected full text, the same process of independent evaluation/extraction was conducted with a previously updated and piloted form.

## Data items

The authors planned to collect data related to the tests, samples included in the original investigation, and the investigation itself including the risk of bias questionnaire.

The data extraction involved collection of information related to the investigation, such as whether it was a multicentre study (the same protocol being executed in different places) and the period of data collection (when inclusion of volunteers were involved from start to finish), sample characteristics, such as the fraction of children (under 18 years old), the sex, mean/median age, minimum and maximum age, fraction of volunteers included from blood banks donors, fraction of volunteers living in either rural or urban areas, and clinical forms (either with indeterminate form or with cardiac form). Information planned for extraction from each of the ELISA tests were tests names and manufacturers, and from each of the PCR tests were whether the primers targeted the kinetoplast-DNA (k-DNA) minicircles or satellite-DNA (sat-DNA) and whether the technique was DNA hybridisation, PCR standard qualitative, nested, or quantitative.

It is important to note that the authors planned in advance to collect many other data; however, they were dropped in the analysis either because there were too many fields with an absence of data or they were too heterogeneous to compile. In the latter case, they are mentioned in the text if appropriate.

## Risk of bias of individual reports

The risk of bias was assessed through a quality assessment tool for diagnostic accuracy studies (QUADAS)-2 (Whiting et al. 2011). Briefly, this tool is designed to assess the quality of primary diagnostic accuracy studies and should be applied in addition to extracting primary data for use in the review. The QUADAS-2 tool consists of four key domains that discuss patient selection, the index test, the reference standard, and the flow of patients through the study and timing of the index tests and reference standard.

Risk of bias is judged as “low”, “high”, or “unclear”. If the answers to all of the signalling questions for a domain are “yes”, then the risk of bias can be judged as low. If any signalling question is answered “no”, the potential for bias exists. The “unclear” category was used when insufficient data are reported to permit a judgement.

Applicability was structured in a way similar to that of the bias sections but do not include signalling questions. Review authors recorded the information on which the judgement of applicability is made and then rate their concern that the study does not match the review question.

## Outcomes

The outcomes of interest were the absolute counts of (i) true positives, (ii) false negatives, (iii) false positives, and (iv) true negatives; these counts were (i) the amount of subjects with Chagas disease and identified by the test as having Chagas disease, (ii) the amount of subjects with Chagas disease and identified by the test as not having Chagas disease, (ii) the amount of subjects without Chagas disease identified by the test as having Chagas disease, and (iv) the amount of subjects without Chagas disease and identified by the test as not having Chagas disease. From these counts, it was possible to estimate by different methods the sensitivity (the fraction of subjects correctly identified with the condition) and specificity (the fraction of subjects correctly identified without the condition). These measures, along with the area under the summary ROC curve (SROC) by the bivariate method, were the outcomes of interest.

The authors also planned to collect data concerning the reliability of the tests. However, due to the absence of data, this outcome was dropped from the analysis.

## Tests of interest

*Index tests* - The first index test is a commercial ELISA for the diagnosis of Chagas disease or ELISA-rec.

The second index test is based on molecular technology, PCR, and its variations, such as commercial or in-house tests, qualitative or quantitative, and with k-DNA or sat-DNA amplification. However, groups of interest were formed as suggested by Schijman et al. (2011) where four different methodologies were suggested to improve PCR performance in an international study.

These methodologies are as follows: (M1) DNA extraction from blood in ethylenediamine tetraacetic acid (EDTA)-guanidine with phenol-chloroform and amplification of sat-DNA using a quantitative PCR, (M2) DNA extraction from blood in EDTA-guanidine with phenol-chloroform and amplification of sat-DNA using conventional qualitative PCR, (M3) DNA extraction from blood in EDTA-guanidine using commercial extraction kits with glass columns and amplification of sat-DNA with quantitative PCR, and (M4) DNA extraction from blood in EDTA-guanidine with phenol-chloroform and amplification of k-DNA (121-122 primers) with hot-start PCR. Finally, and additional group was formed from commercial PCR tests.

## Comparator(s)/control

The desired comparator is the Chagas disease diagnosis as recommended by the Brazilian consensus in which two serological tests of different methodology are conducted in parallel. However, as no reference standard for Chagas disease diagnostic research is widely accepted, research with other reference tests were also included such as latent class analysis. If comparisons were made with more than two serological tests or with parasitological tests, and data using two simultaneous serological tests as reference standard was available as well, the latter was preferred instead.

## Data analysis plan

**Fig. 1:**
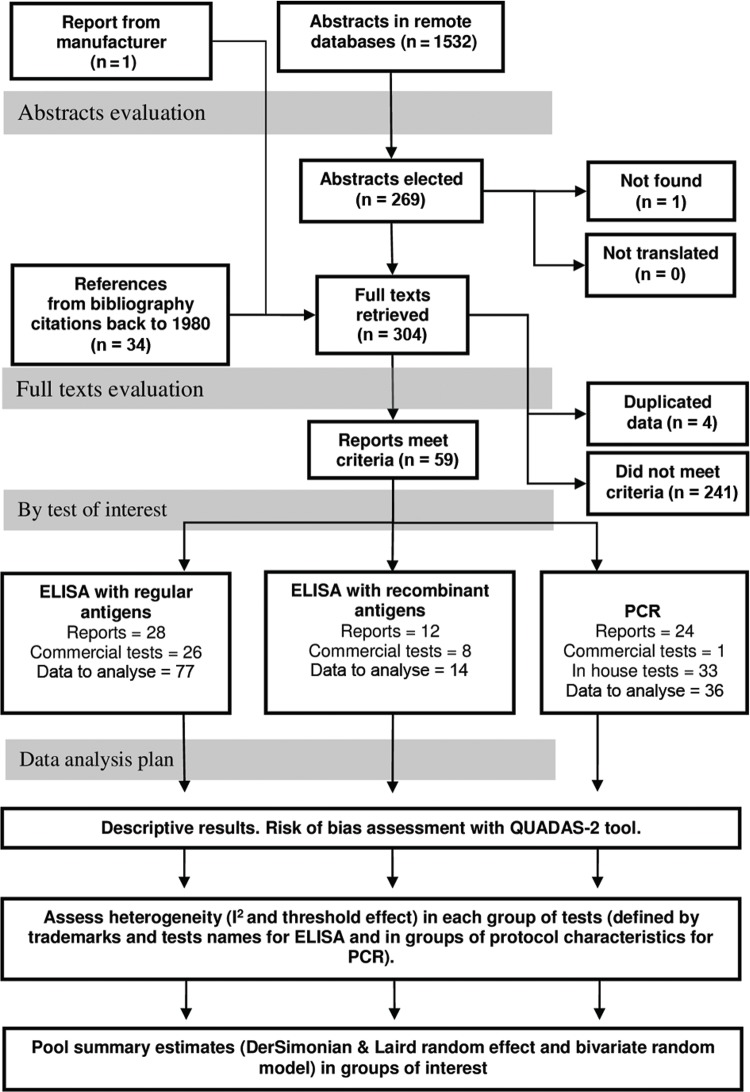
: flowchart of abstracts and full texts evaluation and analysis plan. In each group of reposts, tests, and data to analyse will not match the number of reports included as some investigations have data regarding two or more tests in one or more groups. ELISA: enzyme-linked immunosorbent assay; PCR: polymerase chain reaction; QUADAS: quality assessment tool for diagnostic accuracy studies.

Data synthesis was conducted in each subgroup of interest and was the combination of the trademark and the test name in the case of the ELISA tests. For PCR tests, the groups of interest were whether the tests were commercial or in-house and the variations of PCR procedures. Heterogeneity was explored in and between these groups with the I^2^ and the Cochrane Q test for both sensitivity and specificity (Fig. 1). For all of the groups of trademark and test name with four or more studies, a threshold effect was also explored as a source of heterogeneity. The threshold effect is as a correlation of the test sensitivity and the false positive rate. If a threshold effect is present, a change in test accuracy from different studies is likely to be from differences in the decision thresholds used in each study.

An I^2^ up to 25% was considered low evidence of heterogeneity, 50% or higher was considered high evidence of heterogeneity, and between 25-50% was considered moderate evidence of heterogeneity. A p-value of the Q test lower then 0.10 was also considered evidence of heterogeneity. If there was a conflict of heterogeneity interpretation according to these two tests, the I^2^statistic was considered to be more appropriate. If heterogeneity is high, then one must understand that the pooled summary estimate lacks interpretation.

The authors planned to make the sensitivity (subgroups) analysis according to the sample, tests, and risk of the bias groups; however, the subgroups were often too small to conduct this analysis or small enough to likely mislead the interpretation of the results. The sensitivity and specificity were summarised using two strategies. In the first one, we used a bivariate model proposed by Reitsma (2005) (defined as a linear mixed model with known variances of the random effects). The Cochrane Diagnostic Test Accuracy systematic review group currently recommends this method as the standard approach. Some of its characteristics are considered advantageous: it fits the pair of sensitivity and specificity simultaneously, it returns conservative estimates in the presence of heterogeneity, as correlation is one of its parameters, the presence of the threshold effect reduces the confidence interval, and it allows estimation the SROC; however, it was conducted only in the groups with at least four or more studies. Similar to other regression methods, it has limitations, may not converge, and may mislead interpretation due to convergence problems, especially in small datasets. As an alternative, we used the DerSimonian & Laird random effect (D&L) with a logit transformation (and back-transformation) and the inverse variance method to estimate study weights (as the 2nd method) with all of the groups of interest. All of the analyses were conducted with the R-project statistical package (R Core Team 2015) with libraries meta (Schwarzer 2015) and mada (Doebler 2015).

## Study selection

On the ANVISA website, the authors found 30 records of ELISA tests and no records of commercial PCR tests. Visiting the manufacturers’ websites and by requesting information through their website contact or by phone, we were unable to obtain a return of any technical report during the update period. Nevertheless, the authors looked for possible data of interest in the tests’ technical descriptions and the technical recommendations regarding their use. The majority of these 30 tests did not have any information available regarding the test performance on their websites.

After removing the replicates from the remote bibliographic database strategy, the authors found 1,532 abstracts including the original and the update period (Fig. 1). After including those found through the bibliography search and removing those that did not meet the inclusion criteria or those that met the exclusion criteria, 59 original reports remained for data extraction. As some investigations had data regarding two or more tests, the number of reports, tests, and data to analyse will not match the number of reports included in the review. Data concerning commercial ELISA were extracted from 28 reports (Lorca et al. 1992, Pan et al. 1992, Carvalho et al. 1993, Teixeira et al. 1994,Hamerschlak et al. 1997, Oelemann et al. 1998, Houghton et al. 1999, Leiby et al. 2000, Ferreira et al. 2001,Gadelha et al. 2003, Arrieta et al. 2004, Enciso et al. 2004, Moretti et al. 2004, Pirard et al. 2005,Duarte et al. 2006, Kirchhoff et al. 2006, Malan et al. 2006, Caballero et al. 2007,Tobler et al. 2007, Gorlin et al. 2008, Otani et al. 2009, Remesar et al. 2009,Añez et al. 2010, Flores-Chávez et al. 2010, Barfield et al. 2011, De Marchi et al. 2011, Pereira et al. 2012, Araújo & Berne 2013) (Fig. 1) including 26 different combinations of trademarks and test names. Data from 12 commercial ELISA-rec reports were extracted (Pastini et al. 1994, Gomes et al. 2001, Gadelha et al. 2003, Pirard et al. 2005,Blejer 2006, Chang et al. 2006, Caballero et al. 2007, Ramírez et al. 2009,Remesar et al. 2009, Villagrán et al. 2009, Añez et al. 2010, Pereira et al. 2012, Souza et al. 2012) (Fig. 1), including eight different combinations of trademarks and test names. Finally, data concerning PCR were extracted from 24 reports (Avila et al. 1993, Wincker et al. 1994, 1997, Britto et al. 1995, Espinoza et al. 1996, Junqueira et al. 1996, Carriazo et al. 1998, Chiaramonte et al. 1999, Gomes et al. 1999, Ribeiro-dos-Santos et al. 1999, Castro et al. 2002, Gutierrez et al. 2004, Duarte et al. 2006, Gil et al. 2007, Piron et al. 2007, Fitzwater et al. 2008, Deborggraeve et al. 2009, Ferrer et al. 2009, 20, 2013,Ramírez et al. 2009, Batista et al. 2010, Gilber et al. 2013, Sabino et al. 2013), but only one commercial test was found (Deborggraeve et al. 2009, De Winne et al. 2014). However, the original authors modified the commercial version in two of the studies for research purposes (De Winne et al. 2014).

## Descriptive results

Argentina and Brazil were the countries where most of the studies of serological tests were conducted (Tables I, II). Most of the studies with PCR tests were also conducted in these two countries and in Colombia, Bolivia, and Venezuela (Table III). Very few multicentre investigations were found, and considering the amount of absent information, it was often very hard to know whether the same investigation protocol was applied or just samples from different sources were analysed together. It became clear that the sample description of most of the studies lacked data. The vast majority of the information that was planned to be collected was absent in 60% or more of the reports. Eight (28.6%) of the ELISA reports (Table I), three (25%) of the ELISA-rec studies (Table II), and five (20.8%) of the PCR reports do not have any information regarding the sample description (Table III).

**TABLE I:** Descriptive data of individual enzyme-linked immunosorbent assay test studies

Study	Multicenter	Country	Data collection period	Children proportion (%)	Male proportion (%)	Mean age (years)	Max age (years)	Min age (years)	From blood bank (%)	Living in rural areas (%)	Living in urban areas (%)	With cardiac form (%)	With indeterminate form (%)
Lorca M-1992 (Lorca et al. 1992)	No	Argentina	-	-	-	-	-	-	-	-	-	-	-
Pan AA-1992 (Pan et al. 1992)	Yes	Argentina, Brazil	-	-	-	-	-	-	-	-	-	-	-
Carvalho MR-1993 (Carvalho et al. 1993)	Ignored	Brazil	-	-	-	-	-	-	67	-	-	-	-
Teixeira MGM-1994 (Teixeira et al. 1994)	No	Brazil	-	-	-	-	78	16	-	-	-	-	-
Hamerschlak N-1997 (Hamerschlak et al. 1997)	No	Brazil	-	0	-	-	-	-	100	-	-	-	-
Oeleman WMR-1998 (Oelemann et al. 1998)	No	Brazil	-	-	-	-	-	-	-	-	-	-	-
Houghton RL-1999 (Houghton et al. 1999)	No	Brazil, Equator, USA	-	-	-	-	-	-	-	-	-	-	-
Leiby DA-2000 (Leiby et al. 2000)	No	Brazil	-	-	-	-	-	-	100	-	-	-	-
Ferreira AW-2001 (Ferreira et al. 2001)	No	Brazil	-	-	-	-	-	-	52	-	-	27	-
Gadelha AAM-2003 (Gadelha et al. 2003)	No	Brazil	-	0	-	-	82	21	-	-	-	79	13
Arrieta R-2004 (Arrieta et al. 2004)	Yes	Argentina	2000-2001	100	-	-	14	1	0	-	-	-	-
Enciso C-2004 (Enciso et al. 2004)	No	Colombia	-	-	-	-	-	-	100	-	-	-	-
Moretti E-2004 (Moretti et al. 2004)	No	Argentina	-	-	-	-	-	-	-	-	-	-	-
Pirard M-2005 (Pirard et al. 2005)	No	Bolivia	1998-1999	0	66	31	-	-	100	13	87	-	-
Duarte AMV-2006 (Duarte et al. 2006)	No	Brazil	2000-2002	-	-	-	80	10	-	-	-	48	38
Kirchhoff LV-2006 (Kirchhoff et al. 2006)	Yes	Mexico	1998-2001	0	76	-	-	-	100	-	-	-	-
Malan AK-2006 (Malan et al. 2006)	No	Brazil, USA	-	-	47	49	71	21	45	-	-	50	60
Caballero ZC-2007 (Caballero et al. 2007)	Yes	Brazil, Panama	-	0	60	-	60	18	32	-	-	-	-
Rivetz B-2007^*a*^	Yes	Multicenter	-	-	-	-	-	-	-	-	-	-	-
Tobler LH-2007 (Tobler et al. 2007)	No	USA	-	-	-	-	-	-	96	-	-	-	-
Gorlin J-2008 (Gorlin et al. 2008)	Yes	USA	-	-	-	-	-	-	96	-	-	-	-
Otani MM-2009 (Otani et al. 2009)	Yes	Argentina, Brazil, Colombia, El Salvador, Honduras, Mexico, Nicaragua, Paraguay	2000-2000	0	-	-	-	-	100	-	-	-	-
Remesar MC-2009 (Remesar et al. 2009)	Yes	Argentina	2006-2007	0.6	79	36	-	-	100	-	-	-	-
Añez N-2010 (Añez et al. 2010)	No	Venezuela	-	-	35.7	-	-	-	44.7	-	-	44.7	15.8
Flores Chavez M-2010 (Flores-Chávez et al. 2010)	No	Spain	-	-	-	-	-	-	-	-	-	-	-
Barfield CA-2011 (Barfield et al. 2011)	No	Argentina	-	-	-	-	-	-	-	-	-	-	-
March CR-2011 (De Marchi et al. 2011)	Ignored	Brazil	-	-	-	-	-	-	32.4	-	-	-	-
Araujo AB-2013 (Araújo & Berne 2013)	No	Brazil	-	0	-	-	65	18	100	-	-	0	100

*a*: nonpublished report.

**TABLE II:** Descriptive data for individual enzyme-linked immunosorbent assay recombinant test studies

Study	Multicenter	Country	Data collection period	Children proportion (%)	Male proportion (%)	Mean age (years)	Max age (years)	Min age (years)	From blood bank (%)	Living in rural areas (%)	Living in urban areas (%)	With cardiac form (%)	With indeterminate form (%)
Pastini AC-1994 (Pastini et al. 1994)	No	Argentina	-	-	-	-	-	-	-	-	-	-	-
Gomes YM-2001 (Gomes et al. 2001)	No	Brazil	-	-	-	-	76	5	-	-	-	-	-
Gadelha AAM-2003 (Gadelha et al. 2003)	No	Brazil	-	-	-	-	82	21	-	-	-	79	13.39
Blejer JL-2006 (Blejer 2006)	No	Argentina	1995-2003	-	-	-	-	-	100	-	-	-	-
Pirard M-2005 (Pirard et al. 2005)	No	Bolivia	1998-1999	0	66	31	-	-	100	13	87	-	-
Chang CD-2006 (Chang et al. 2006)	No	USA	-	-	-	-	-	-	100	0	0	-	-
Caballero ZC-2007 (Caballero et al. 2007)	Yes	Brazil, Panama	-	0	60	-	60	18	32	-	-	-	-
Ramírez JD-2009 (Ramírez et al. 2009)	No	Colombia	-	0	-	-	-	-	-	-	-	100	0
Remesar MC-2009 (Remesar et al. 2009)	Yes	Argentina	2006-2007	0	79	36	-	-	100	-	-	-	-
Villagrán ME-2009 (Villagrán et al. 2009)	Yes	Mexico	-	-	-	-	-	-	-	-	-	-	-
Añez N-2010 (Añez et al. 2010)	No	Venezuela	-	-	35.7	-	-	-	44.7	-	-	44.7	15.8
Souza RM-2012 (de Souza et al. 2012)	No	Brazil	-	-	-	-	-	-	-	-	-	-	-

**TABLE III:** Descriptive data of individual polymerase chain reaction test studies

Study	Multicenter	Country	Data collection period	Children proportion (%)	Male proportion (%)	Mean age (years)	Max age (years)	Min age (years)	From blood bank (%)	Living in rural areas (%)	Living in urban areas (%)	With cardiac form (%)	With indeterminate form (%)
Avila HA-1993 (Avila et al. 1993)	No	Brazil	-	-	-	-	84	13	15	-	-	-	-
Wincker P-1994 (Wincker et al. 1994)	No	Brazil	1992-1992	-	-	-	-	-	0	-	-	-	-
Britto C-1995 (Britto et al. 1995)	No	Brazil	-	-	-	-	-	-	-	-	-	-	-
Espinoza AG-1996 (Espinoza et al. 1996)	No	Bolivia	-	77	0	-	-	-	-	-	-	-	-
Junqueira ACV-1996 (Junqueira et al. 1996)	No	Brazil	1993-1994	-	-	-	-	-	-	-	-	-	-
Wincker P-1997 (Wincker et al. 1997)	No	Bolivia	-	100	-	7	15	1	0	100	0	-	-
Carriazo CS-1998 (Carriazo et al. 1998)	No	Argentina	-	-	-	-	-	-	-	-	-	-	-
Chiaramonte MG-1999 (Chiaramonte et al. 1999)	No	Argentina	-	-	-	-	-	-	-	-	-	-	-
Gomes ML-1999 (Gomes et al. 1999)	No	Brazil	-	-	46	46	76	11	-	-	-	-	-
Ribeiro-dos-Santos G-1999 (Ribeiro-dos-Santos et al. 1999)	No	Brazil	-	-	-	-	-	-	100	-	-	-	-
Castro AM-2002 (Castro et al. 2002)	No	Brazil	-	0	42	51	88	23	-	-	-	-	-
Gutierrez R-2004 (Gutierrez et al. 2004)	No	Colombia	-	-	-	-	-	-	-	-	-	-	-
Duarte AMV-2006 (Duarte et al. 2006)	No	Brazil	2000-2002	-	-	-	80	10	-	-	-	48	38
Gil J-2007 (Gil et al. 2007)	No	Colombia	-	0	-	-	65	18	-	-	-	46	10
Piron M-2007 (Piron et al. 2007)	No	Spain	2000-2004	1	-	-	-	1	-	-	-	-	-
Fitzwater S-2008 (Fitzwater et al. 2008)	No	Peru	2006-2007	-	0	24	45	13	0	-	-	-	-
Deborggraeve S-2009 (Deborggraeve et al. 2009)	Ignored	Belgium	2000-2004	0	-	-	-	-	35	-	-	11	-
Ferrer E-2009 (Ferrer et al. 2009)	No	Venezuela	-	-	-	-	-	-	-	-	-	-	-
Ramírez JD-2009 (Ramírez et al. 2009)	No	Colombia	-	0	-	-	-	-	-	-	-	100	0
Batista AM-2010 (Batista et al. 2010)	No	Brazil	1999-2007	-	-	-	-	-	0	-	-	-	13.5
Ferrer E-2013 (Ferrer et al. 2013)	No	Venezuela	-	-	-	-	-	-	27.8	-	-	-	-
Gilber SR-2013 (Gilber et al. 2013)	No	Brazil	-	-	-	55.3	89	10	-	-	-	-	-
Sabino EC-2013 (Sabino et al. 2013)	Yes	Brazil, Honduras, USA	2007-2012	0	-	-	-	-	100	-	-	-	-
Winne KD-2014 (De Winne et al. 2014)	Yes	Argentina, Chile, Spain	2004-2012	-	-	-	-	-	29.2	-	-	-	-

The authors planned to collect data regarding the serological tests’ antigens, whether the antigens were purified or not, recommended decision threshold, and how the decision thresholds were estimated, but the tests’ descriptions were often so poor that the desired information was not available. When looking for accuracy data on the manufacturers’ websites, the authors were sometimes able to read the instructions to conduct the tests. When that was the case, the serology decision threshold was always dependent of internal controls and formulas such as “the arithmetic mean of negative and positive controls” are frequent. This indicates that each time someone runs the test, a new decision threshold may arise and its basis is solely on analytic information.

The reference standard was also highly heterogeneous among all of the studies. Twenty-one percent of all of the investigations did not even apply the same reference standard to classify those with and without Chagas disease. Only 22% of the studies applied a reference standard similar to the Brazilian consensus in which two serological tests must be either positive or negative, and 3.5% used latent class analysis. The remaining studies applied reference standards such as one serological test (21%), two positives out of three tests (21%), ignored (14%) and unusual combinations of different serological tests (e.g., western blot, haemagglutination, and two ELISAs), and combinations of serological tests with parasitological tests or partial verification of the sample.

## Risk of bias assessment

**Fig. 2:**
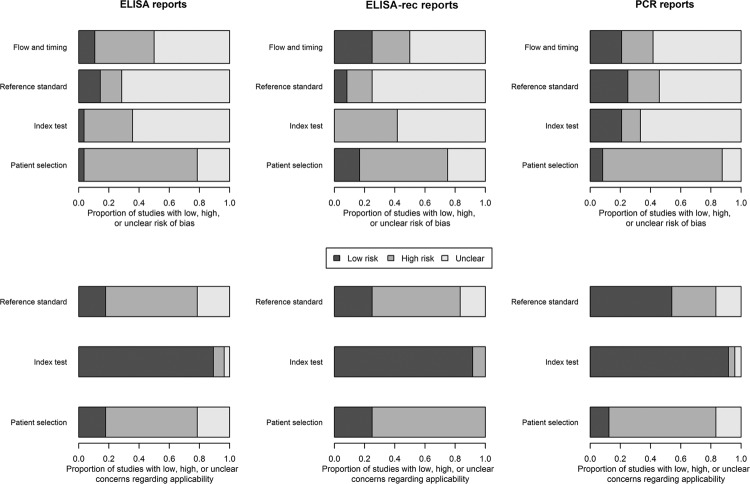
: risk of bias assessment by topic regarding study design/conducting and test applicability. ELISA: enzyme-linked immunosorbent assay; PCR: polymerase chain reaction.

The risk of bias assessment made through the QUADAS-2 tool is shown in Fig. 2. No more than 30% of studies were classified as “low risk” of bias in any of the evaluated dimensions or in any of the three tests. In the “patient selection” dimension, the majority of the studies were classified as having a high risk of bias. This occurred because most of the selected volunteers/samples were a case mix of unexposed to Chagas disease or samples from patients with other diseases where the suspicion of Chagas disease was unlikely. The remaining assessment of the risk of bias, “flow and timing”, “reference standard”, and “index tests” had the majority of studies classified as “unclear”. Again, this is an indicator of the amount of the absence of data and did not permit the authors to conduct appropriate classification for review purposes.

The risk of bias results given the concerns about applicability is a little different from the risk of bias results from the research design/conduction. The majority of the reports of ELISA and the PCR tests were classified as “low risk” in the index test dimension. Again, the majority of the studies were classified as “unclear risk” concerning the applicability in the “reference standard”, and “patient selection” topics in all three serological and molecular tests (Fig. 2).

**Fig. 3:**
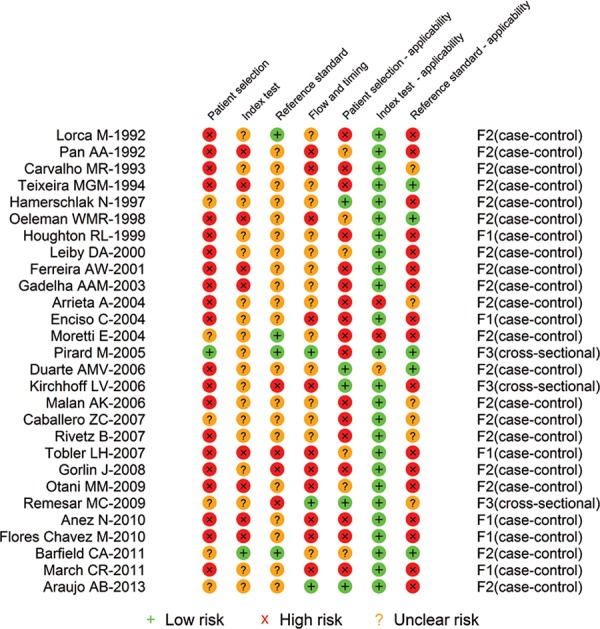
: quality assessment tool for diagnostic accuracy studies risk of bias of individual studies of enzyme-linked immunosorbent assay tests.

**Fig. 4:**
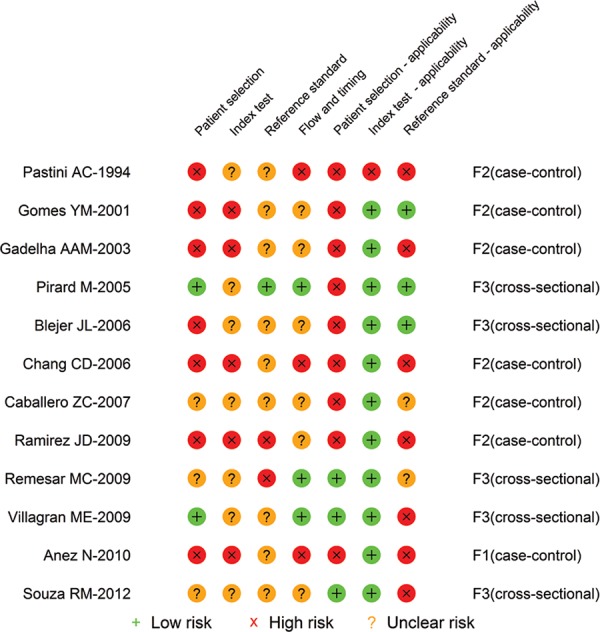
: quality assessment tool for diagnostic accuracy studies risk of bias of individual studies of enzyme-linked immunosorbent assay tests with recombinant antigens.

**Fig. 5:**
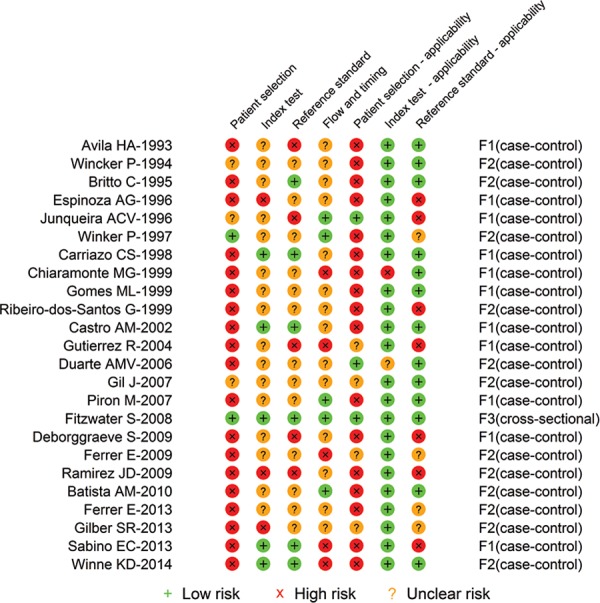
: quality assessment tool for diagnostic accuracy studies risk of bias of individual studies of polymerase chain reaction tests.

Looking at the individual reports’ classifications about their risk of bias for the ELISA (Fig. 3), ELISA-rec (Fig. 4), and PCR (Table IV), it is observed that the number of classifications of “low risk” within each study is heterogeneous and ranges from 0-5 in the ELISA and ELISA-rec reports and from 1-7 in the PCR reports. The majority of ELISA (Fig. 3) and ELISA-rec (Fig. 4) studies do not have more than two topics assessed as “low risk” of bias, whereas in the PCR studies, the majority have no more than three topics assessed as “low risk”. Only one PCR study had all seven topics assessed as “low risk” of bias (Fig. 5).

**TABLE IV:** Summary estimates from bivariate model of different polymerase chain reaction (PCR) methodologies studied at least four times

Test	Measure	Estimate	95% CI.lb	95% CI.ub
M4	Sensitivity	0.457	0.091	0.877
	Specificity	0.958	0.858	0.989
	AUC SROC	0.94	-	-
	Threshold effect	0.342	-0.554	0.871
None	Sensitivity	0.654	0.493	0.786
	Specificity	0.972	0.914	0.991
	AUC SROC	0.919	-	-
	Threshold effect	0.373	-0.036	0.675

accuracies without significant threshold effect are not interpretable. AUC SROC: area under the summary receiver operating characteristic curve; CI.lb: confidence interval lower bound; CI.ub: confidence interval upper bound; M4: DNA extraction from blood in ethylenediamine tetraacetic acid-guanidine with phenol-chloroform, amplification of kinetoplast-DNA (121-122 primers) with hot-start PCR; none: none of the improvements propose by Schijman.

Studies classified as development Phase 3 or later are considered the most suitable to give results for decision making because they are those which most resemble clinical practice (Haynes & You 2009); however, few studies were classified as development Phase 3 in this review. Only three ELISA studies, five ELISA-rec studies, and one PCR study were classified as Phase 3.

## Heterogeneity and summary estimates

With very few exceptions, the ELISAs’ sensitivity and specificity point estimates are above 90% in all of the studies (Supplementary Figure 1), and this occurs with ELISA-rec (Supplementary Figure 2) as well. This finding is compatible with the sensitivity and specificity point estimate provided by the manufacturers, although, in most cases, the manufacturers clearly provided higher accuracy estimates than the academic studies (Table V). What stands out is that 56% of the manufacturers do not explicitly provide accuracy information regarding commercial tests on their websites or in the test documentation (Table V), and for the tests where the information is available, there is neither an estimate of confidence intervals nor information on how they reached the accuracy results. Only rarely do manufacturers provide a bibliography for further reading on their website, which may contain some information about study design, conducting the study, and the accuracy results.

**TABLE V:** Country, manufacturer, and accuracies of commercial enzyme-linked immunosorbent assay (ELISA) and ELISA recombinant tests provided by the manufacturers on their websites

Country	Manufacturer	Test	Sensitivity	Specificity
USA	Abbott Laboratories	Abbott ESA Chagas	1.000	0.991 or 0.925
USA	Abbott Laboratories	Abbott PRISM Chagas	0.984	0.987
Italy	Adaltis	EIAgen Trypanosoma cruzi Ab	NA	NA
Spain	Biokit	Bioelisa CHAGAS	1.000	0.974-0.995
Brazil	Bio-Manguinhos	Bio-Manguinhos EIA	NA	NA
Brazil	Bio-Manguinhos	Bio-Manguinhos EIA recombinant	NA	NA
France	bioMérieux SA	ELISA cruzi	NA	NA
Chile	BiosChile	Test ELISA Chagas III	1.000	1.000
Spain	BLK Diagnostics	ELISA BLK	NA	NA
Australia	Cellabs Pty Ltd	Cellabs T. cruzi IgG CELISA	0.98	0.98
Belgium	Coris BioConcept	T. cruzi OligoC-TesT	1.00	1.00
Brazil	Ebram Produtos Laboratoriais Ltda	CHAGAS ELISA	1.00	1.00
Brazil	Embrabio Empresa Brasileira de Biotecnologia SA	HEMOBIO-CHAGAS	NA	NA
Argentina	Gador SA	Dia Kit Bio-Chagas	NA	NA
Ireland	GenCell Biosystems	cruziTEST ELISA	NA	NA
USA	Gull Laboratories Inc/Meridian Bioscience Inc	Gull ELISA	NA	NA
USA	Hemagen Diagnostics Inc	Hemagen Chagas’ Kit	NA	NA
Paraguay	Research Institute for Health Sciences /National University of Asunción	Chagas Test ELISA	NA	NA
USA	IVD Research Inc	IVD ELISA	NA	NA
Argentina	Laboratório Lemos SRL	BIOZIMA CHAGAS ELISA	NA	NA
Argentina	Laboratório Lemos SRL	Chagatek	NA	NA
Argentina	Laboratório Lemos SRL	PATH-Lemos rapid test	NA	NA
USA	Meridian Bioscience Inc	CHAGAS IgG EIA	NA	NA
Scotland	Omega Diagnostics Ltd	PATHOZYME CHAGAS	0.983	0.985
USA	Orgenics Ltd/Alere Inc	ImmunoComb^®^ II Chagas Ab Kit	1.00	0.983
USA	Ortho-Clinical Diagnostics Inc	ORTHO T. cruzi ELISA Test System	1.00	0.999
USA	Siemens Healthcare	Siemens IMMULITE CHAGAS IgG	NA	NA
Brazil	Symbiosys	ANTI-CHAGAS SYMBIOSYS	1.00	0.993
Argentina	Wiener Laboratórios	Chagatest ELISA lisado	1.00	0.992
Argentina	Wiener Laboratórios	Chagastest ELISA recombinante v.4.0	1.00	0.996

NA: not available.

Again, one must understand that the excess of heterogeneity turns the summary estimate into a noninterpretable status, although this excess may be accounted for in a bivariate model approach if the threshold effect is present. Overall, the evidence of heterogeneity was high except in the Research Institute for Health Sciences (IICS) of the National University of Asunción - Chagas Test ELISA, and the Bio-Manguinhos enzyme immunoassay (EIA) Recombinant studies, where there is a low evidence of heterogeneity in both sensitivity and specificity. D&L pools the sensitivity and specificity separately; therefore, it is possible to detect different levels of heterogeneity in sensitivity and specificity in the same test. This is the case of the bioMérieux - BioElisacruzi, Lemos - BIOZIMA CHAGAS ELISA, and Biokit - BIOELISA CHAGAS tests, where there is low evidence of heterogeneity in sensitivity and high evidence of heterogeneity in specificity. This also happened with the Lemos - Chagatek, and Wiener - CHAGAS TEST tests, in which there is moderate evidence of heterogeneity in sensitivity and high evidence of heterogeneity in specificity. Either there was a high evidence of heterogeneity in both sensitivity and specificity in the remaining tests or they were studied just once, in which case it was not possible to estimate heterogeneity.

Only two ELISA-rec tests were studied at least twice to allow the heterogeneity estimates (Supplementary Figure 2). The Bio-Manguinhos EIA Recombinant was studied twice and the evidence of heterogeneity in both sensitivity and specificity was low. The Wiener CHAGAS TEST recombinant was studied seven times and evidence of heterogeneity was high in both sensitivity and specificity.

For where the evidence of heterogeneity was low (i.e., the Bio-Manguinhos EIA Recombinant and IICS of National University of Asunción - Chagas Test ELISA), the summary sensitivity was 0.98 and 0.97, and the summary specificity was 1.00 and 0.99, respectively (Supplementary Figures 1,2). The remaining D&L summary estimates of the ELISA tests (Supplementary Figure 1) and ELISA-rec tests (Supplementary Figure 2) have limited interpretation due to their heterogeneity.

The Abbott Laboratories - ABBOTT CHAGAS ELISA, Gull - ELISA, and Lemos - Chagatek tests had almost identical summary pooled estimate results by bivariate and D&L models; however, a threshold effect was detected. The bivariate model summary estimate (Table VI) showed a slightly lower accuracy than the D&L estimate with narrower confidence intervals. In this case, they were considered more appropriate and result in interpretable summary estimates. In addition, the presence of the threshold effect turns the area under the ROC curve into an attractive accuracy measure for performance interpretation.

**TABLE VI:** Summary estimates from bivariate model of enzyme-linked immunosorbent assay tests studied at least four times

Manufacturer/test	Measure	Estimate	95% CI.lb	95% CI.ub
Abbott Laboratories/ Abbott Chagas Elisa	Sensitivity	0.95	0.931	0.964
	Specificity	0.985	0.963	0.994
	AUC SROC	0.971	-	-
	Threshold effect	0.732^*a*^	0.056	0.948
bioMérieux SA/ BioELISAcruzi	Sensitivity	0.99	0.976	0.995
	Specificity	0.961	0.898	0.986
	AUC SROC	0.987	-	-
	Threshold effect	0.231	-0.715	0.878
BiosChile/ Test ELISA Chagas	Sensitivity	0.977	0.902	0.995
	Specificity	0.987	0.908	0.998
	AUC SROC	0.988	-	-
	Threshold effect	0.444	-0.721	0.953
Gull Laboratories Inc/ ELISA	Sensitivity	0.966	0.88	0.991
	Specificity	0.935	0.785	0.983
	AUC SROC	0.981	-	-
	Threshold effect	-0.986^*a*^	-0.999	-0.793
Laboratório Lemos SRL/ Chagatek	Sensitivity	0.986	0.965	0.994
	Specificity	0.922	0.757	0.978
	AUC SROC	0.984	-	-
	Threshold effect	-0.857	-0.978	-0.292
Orgenics Ltd/ ImmunoComb^®^ II Chagas Ab Kit	Sensitivity	0.991	0.955	0.998
	Specificity	0.975	0.948	0.988
	AUC SROC	0.989	-	-
	Threshold effect	-0.412	-0.845	0.348
Ortho-Clinical Diagnostics Inc/ T. cruzi ELISA	Sensitivity	0.992	0.949	0.999
	Specificity	0.991	0.913	0.999
	AUC SROC	0.989	-	-
	Threshold effect	0.454	-0.715	0.954
Wiener Laboratórios/ Chagastest recombinante	Sensitivity	0.937	0.877	0.969
	Specificity	0.99	0.976	0.996
	AUC SROC	0.987	-	-
	Threshold effect	0.505	-0.4	0.911

*a*: significant threshold effect. Accuracies without significant threshold effect are not interpretable; AUC SROC: area under the summary receiver operating characteristic curve; CI.lb: confidence interval lower bound; CI.ub: confidence interval upper bound.

The Ortho-Clinical Diagnostics - T. cruzi ELISA, Lemos - BIOZIMA CHAGAS ELISA, bioMérieux - BioElisacruzi, BiosChile - Test ELISA Chagas, Wiener - CHAGASTEST, and Wiener - CHAGASTEST recombinant tests had different evidence of heterogeneity in sensitivity and specificity, and no threshold effect was detected. The pooled summary estimates of both models are almost identical; thus, neither the area under the SROC curve nor the sensitivity and specificity estimates were considered interpretable. The three tests with four or more studies that are not in Supplementary Figure 1 or Table VI (i.e., the Embrabio - HEMOBIO CHAGAS, Lemos - BIOZIMA CHAGAS ELISA, and Wiener - CHAGASTEST test) did not converge in the bivariate model and are not presented on purpose.

It was expected that the PCR results would be much more heterogeneous than the serology studies’ results. The authors were able to find only two reports with a commercial version of PCR, and the authors modified the test in one of them. Although in this review the PCR tests were grouped according to the specific group of methods previously suggested for PCR improvement, the authors are aware that there is no identical PCR test protocol among all of the reports found. Once more, the bivariate model (Table IV) returns similar summary statistics when compared to the D&L model (Supplementary Figure 3, Table IV). Both summary pooled estimates are not interpretable due the amount of heterogeneity and the differences of protocols (Supplementary Figure 3). Similar to the serological studies, the summary estimates by the bivariate model (Table IV) are also difficult to interpret.

The main results of this research are: (i) the lack of information regarding all dimensions is significantly high including study sample description, heterogeneous reference standards and the tests’ key issues (e.g., the decision thresholds and antigens used), (ii) the fraction of “low risk” of bias is low in almost all of the dimension groups in the risk of bias assessment and only one PCR study was classified as low risk of bias in all of the dimensions, (iii) there was evidence of moderate or high heterogeneity in most cases of the serological tests, but in two tests a threshold effect was evident, and (iv) heterogeneity was even more evident in the PCR studies. This amount of heterogeneity in the PCR tests was expected because there are no identical protocols and only one commercial test was found.

In practice, there are several differences in clinical and pre-clinical diagnostic test validation phases. These phases are also known as “laboratory validation” and “clinical validation”. Didactically, the latter is divided into three-five phases, similar to clinical trials (Haynes & You 2009). These phases of the clinical validation refer to an increasing maturation of the results to a straightforward clinical interpretation. Usually, there is an overestimation of the test accuracy in the early phases (Haynes & You 2009).

Similar to the period of the first review (1980-2009), in the update period, basic science (parasitology and immunology) journals and their authors were the most prevalent. This could be a good explanation for the amount of missing data regarding clinical sample description and study design, as the research in these basic areas is more concerned with the tests themselves and less likely to manage clinical research design, conduction, and interpretation. Nevertheless, data from the update period (from 2009-2014) showed a clearly increasing awareness in academia regarding differences of clinical and laboratory validation and laboratory researchers seem to be increasingly devoting more efforts to strictly laboratory validation (Schijman et al. 2011).

Key issues for clinical decision making, such as the definition of reference standards and decision thresholds, are usually defined in analytical ways; therefore, there is less practical and difficult to interpret scenarios from the clinical point of view. There is evidence that using a reference standard similar to the index test, will overestimate the index test accuracy (Lijmer et al. 1999, Rutjes et al. 2006), which probably occurs with the ELISA studies. Similarly, using an imperfect reference standard that poorly classifies the subjects without the target condition will underestimate the index test specificity, which probably occurs in some the PCR studies. In addition, there is evidence of an overestimation of test accuracy when the decision threshold is estimated with the data where accuracy is also estimated (Whiting et al. 2011). This review shows evidence that the importance of decision thresholds is still neglected in this field.

Diagnostic test development and validation are influenced by regulatory agency legislation. Usually, the regulations to register and commercialise diagnostic tests are less restricted than those for medicines, vaccines, or invasive devices, which is probably due to safety issues (Hinman et al. 2006). For example, the current ANVISA regulation states that diagnostic tests or devices conducted in biological samples in artificial containers (outside the patient) do not need a technical report with efficacy/accuracy or safety estimates for registration. There is evidence that research investments for development of diagnostic tests are limited in relation to drug development due to the characteristics of its life cycle. A test/platform development and validation frequently involves the need of periodic improvements and often it is considered a higher risk product for industry compared to medicines (Hinman et al. 2006). The available tests on the market are likely to have questionable and overestimated accuracies when required improvements are not conducted.

Unfortunately, the risk of bias assessment showed a bias susceptibility mainly in the patient selection dimension. Nevertheless, the risk is either unknown or high in almost every dimension. The bias may be in any magnitude and in both directions, and it seems to be a common issue in diagnostic test investigations and diagnostic tests’ accuracy reviews (Leeflang et al. 2007). However, there is evidence supporting that the presence of bias leads to tests’ accuracies overestimation (Lijmer et al. 1999), which converges with the reasoning of overestimated accuracies discussed above. To reduce the risk of bias in diagnostic test research, future research should focus attention on conducting and reporting on the following topics: research should be conducted at clinical settings where patients are included consecutively and their main inclusion criteria should be chronic Chagas disease suspicion. In addition, the index test and reference test must be conducted independently and blinded to each other. Their description should be detailed enough to allow others to reproduce the methods. Usually, these topics characterise and are more often present for Phase 3 diagnostic test research.

Heterogeneity is defined as a variation of the study results beyond random occurrence (Egger et al. 2001). It may be due to sample, design, how the study was conducted, and reporting differences. It was stated before that heterogeneity is a rule in systematic reviews of diagnostic tests’ accuracies (Buntinx et al. 2009); nevertheless, it is not always possible to identify heterogeneity sources with subgroup analysis. For the same reason, the bivariate model has been recommended as one analysis approach to work around this issue.

A particular source of heterogeneity in diagnostic test research is the threshold effect. Different observed accuracies of the same test may be determined by the use of different decision thresholds. It is likely that in many cases in this review, the determined heterogeneity is a mix of bias and the threshold effect, as the ELISA tests thresholds are estimated using internal controls by a variety of criteria, and its values may vary from run to run and even in the same test. As the original studies are using different thresholds, it is very hard to tie the summary estimates to a particular threshold, even if threshold effect is detected and the estimated summary accuracy considers it. Therefore, one cannot make recommendations about which threshold should be used in this particular test. The threshold effect is one of the parameters in the bivariate model and it therefore accounts for this source of heterogeneity. In the presence of heterogeneity and the absence of a threshold effect, the bivariate approach estimates larger variances and thus wider confidence intervals. Therefore, it returns a conservative interpretation in this situation. However, it requires a large number of studies to accurately estimate the point summary sensitivity and specificity.

High evidence of heterogeneity among the PCR tests was expected. However, less heterogeneity of recent studies was also expected where available technology makes the tests less operator-dependent. Currently, the costs of PCR tests are quickly decreasing, and the equipment to conduct fast and automated molecular tests are becoming widely available. It is likely that the amounts of differences in PCR protocols are due to fast adoption of new techniques/methodologies/equipment without sufficient evidence concerning the clinical applicability of previous generations of protocols. Based on an international study (Schijman et al. 2011), four different approaches were recommended as the best analytical methods. Unfortunately, not all of these four recommendations were used in clinical research for chronic Chagas diagnosis, and the currently there is high evidence of heterogeneity. Therefore, it not yet possible to confirm that any of these four recommendations perform better in clinical settings.

There are some settings where PCR can be quite useful, such as an assessment of a cure after trypanocidal treatment, identification of Chagas disease reactivation after transplant, and diagnosis of acute infection. Reference/research laboratories may be able to apply PCR using specific algorithms that sometimes combine a few PCR tests or PCR with serological tests for several purposes. This practice is particularly interesting in epidemiological investigations as PCR is currently the only technique that offers genotype capabilities.

One issue about the PCR test is that many believe that it has 100% specificity due to the DNA’s analytical specificity. However, in this review we found many reports with a PCR specificity point estimate lower than 100%. This phenomenon may occur because it may indeed not have 100% specificity, as test contamination may raise false positive results, and specificity underestimation may occur as the reference standard serology does not correctly identify those without Chagas disease. In the latter case, there is some discussion in literature regarding seronegative cases of chronic Chagas disease (Salomone et al. 2003,Batista et al. 2010, Blum-Domínguez et al. 2011). However, there is an inherent limitation of PCR tests imposed by the disease, which is that parasitic presence in the blood stream is required.

A PCR assay needs a small amount of specific DNA target sequences, as a template of the parasite, in peripheral blood samples for an appropriate amplification. This reasoning indicates that PCR assays are promising tools. However, this analytical sensitivity was not transposed to clinical sensitivity as shown in this review. There is evidence that parasitaemia is low in the chronic phase (Castro et al. 1999, Castro & Prata 2000, de Freitas et al. 2011). Therefore, the accuracy of PCR is compromised by an unknown behaviour of *T. cruzi* parasitaemia in the chronic phase, where periods of detectable parasitaemia are not predictable. One way to improve the PCR performance for chronic Chagas disease diagnosis would be to detect a predictable pattern of the parasite in the blood stream and use this pattern to choose a more convenient moment to collect blood samples. A possible workaround on this limitation is to collect a series of blood samples because it may increase the probability of identifying the parasitic DNA in at least one of the samples.

Several studies use repetitive regions as a strategy for Chagas disease molecular diagnosis in an attempt to overcome the low sensitivity of the test, although there is no evidence pointing to this direction so far. Other suggestions to improve PCR performance are: parasite concentration, to use two or more PCR assays (Fitzwater et al. 2008, Qvarnstrom et al. 2012) or to use two or more primers simultaneously. Although there is no consistent evidence that these procedures improve clinical PCR performance for chronic Chagas disease diagnosis, this later strategy was previously applied for development of a diagnostic assay of other neglected tropical diseases using loop-mediated isothermal amplification (Dinzouna-Boutamba et al. 2014).

## Concluding remarks

In conclusion, evidence supports that commercial ELISA and ELISA-rec tests’ known accuracies are probably biased and overestimated. Therefore, to improve diagnostic investigation, studies of test accuracies that are less susceptible to bias are needed. This will probably occur when key issues are adopted, such as: consecutive inclusion of suspected subjects, a reference standard for chronic Chagas disease diagnosis widely accepted in the scientific community and research with later-phase designs. The current recommendation to use two simultaneous serological tests for chronic Chagas disease diagnosis is neither supported by the accuracies found in research papers nor the accuracies provided by the manufacturers. However, this recommendation may be supported by evidence of the heterogeneity of the available tests’ accuracies, the absence of key data in the studies, the likelihood of overestimated accuracies, and perhaps, the prevalence of inconclusive results during clinical investigations.

PCR test usefulness is debatable and health care providers should not order it as a routine test for chronic Chagas disease diagnostic investigation. The single existent commercial test is not widely available, and its accuracy provided by the manufacturer is likely overestimated. The several different in-house protocols lead to a wide range of sensitivity and specificity. PCR’s sensitivity is probably limited by the characteristics of the disease itself. Research/reference centres that are able to conduct PCR and perform it in selected cases, either alone or in combination with serology, are likely to bring some benefit to chronic Chagas disease diagnosis. This practice may be supported by the fact that PCR tests have the potential to detect seronegative cases. However, the performance of this combination and the frequency of these seronegative cases are unknown so far.
